# Characterization of Nodal/TGF-Lefty Signaling Pathway Gene Variants for Possible Roles in Congenital Heart Diseases

**DOI:** 10.1371/journal.pone.0104535

**Published:** 2014-08-11

**Authors:** Xia Deng, Jing Zhou, Fei-Feng Li, Peng Yan, Er-Ying Zhao, Ling Hao, Kai-Jiang Yu, Shu-Lin Liu

**Affiliations:** 1 Genomics Research Center (one of the State-Province Key Laboratory of Biopharmaceutical Engineering, China), Harbin Medical University, Harbin, China; 2 Intensive Care Unit, the Second Affiliated Hospital of Harbin Medical University, Harbin, China; 3 Department of Colorectal Surgery, the Second Affiliated Hospital of Harbin Medical University, Harbin, China; 4 Department of Oncology, the Fourth Affiliated Hospital of Harbin Medical University, Harbin, China; 5 Department of Microbiology and Infectious Diseases, University of Calgary, Calgary, Canada; Institut Jacques Monod, France

## Abstract

**Background:**

Nodal/TGF-Lefty signaling pathway has important effects at early stages of differentiation of human embryonic stem cells in directing them to differentiate into different embryonic lineages. LEFTY, one of transforming growth factors in the Nodal/TGF-Lefty signaling pathway, plays an important role in the development of heart. The aim of this work was to find evidence on whether *Lefty* variations are associated with congenital heart diseases (CHD).

**Methods:**

We sequenced the *Lefty* gene for 230 Chinese Han CHD patients and evaluated SNPs rs2295418, rs360057 and g.G169A, which are located within the translated regions of the genes. The statistical analyses were conducted using Chi-Square Tests as implemented in SPSS (version 13.0). The Hardy-Weinberg equilibrium test of the population was carried out using online software OEGE, and multiple-sequence alignments of LEFTY proteins were carried out using the Vector NTI software.

**Results:**

Two heterozygous variants in *Lefty1* gene, g.G169A and g.A1035C, and one heterozygous variant in *Lefty2* gene, g.C925A, were identified. Statistical analyses showed that the rs2295418 (g.C925A) variant in *Lefty2* gene was obviously associated with the risk of CHD (P value = 0.016<0.05). The genotype frequency of rs360057 (g.A1035C) variant in *Lefty1* gene was associated with the risk of CHD (P value = 0.007<0.05), but the allele frequency was not (P value = 0.317>0.05).

**Conclusions:**

The SNP rs2295418 in the *Lefty2* gene is associated with CHD in Chinese Han populations.

## Introduction

Congenital heart diseases (CHD) are a group of common and complex illnesses with high morbidity and mortality. Despite the enormous advances in surgical treatments over the past decades, the genetic etiology is still largely unknown [1]. The incidence of moderate and severe forms of CHD is about 6/1,000 of live births. If tiny muscular ventricular septal defects and other trivial lesions are included, the total incidence is about 75/1,000 of live births [2]. For the CHD patients, about one percent would require intervention [3] and about thirteen percent show recognizable chromosomal variants [4]. Most adult CHD patients are predisposed to cardiac complications, such as coronary heart diseases, arrhythmias or heart failure [5]. Although extensive genetic studies and high-resolution technologies have revealed the genetic defects in many familiar and sporadic CHD cases [6], [7], the genetic abnormalities in the majority of CHD patients remain largely unknown.

In the embryonic development, heart is the first formed organ, strictly controlled by gene regulatory networks, involving transcription factors, signaling pathways, epigenetic factors, and miRNAs [8], [9]. During the last few decades, a variety of CHD-causing gene mutations have been identified, such as those in *CITED2* [10], *CFC1* [11], *GATA4* [12] and *TBX1* [13]. These genes play critical roles in cardiac development; mutations in these genes lead to cardiovascular malformations and contribute to CHD [14]. Human embryonic stem (HES) cells may differentiate to various cell types and develop to different embryonic lineages, including those of ectoderm (neurons and epidermal cells), endoderm (hepatocytes and pancreatic cells), and mesoderm (muscle cells and cardiomyocytes cells) under the control of certain factors[15]. LEFTY negatively regulates the Nodal/TGF-Lefty signaling pathway [16] and inhibits cellular proliferation and differentiation [17], [18]. It has been shown that when the Nodal/TGF-Lefty pathway goes wrong, serious malignant transformation may occur. In malignant melanoma cells, for example, LEFTY inhibits the malignant properties of melanoma cells [19] [20], [21]. During the early differentiation of HES cells, LEFTY is expressed in a subset of cells, playing an important role in mesodermal cell differentiation [22]. The Nodal/TGF-Lefty signaling pathway also has an important effect in early stages of HES cell differentiation, directing specific cells into different embryonic lineages. LEFTY, as one of the important transforming growth factors in the Nodal/TGF-Lefty signaling pathway, inhibits the signaling of NODAL, which may play an important role in the development of heart.

To elucidate possible associations of *Lefty* genes with CHD, we analyzed the transcribed region and splicing sites of the *Lefty1* and *Lefty2* genes and compared the *Lefty* gene sequences between 230 Chinese Han CHD patients and 263 controls. We found that the rs2295418 (g.C925A) variant in the *Lefty2* gene was closely associated with the risk of CHD.

## Materials and Methods

### The study population

For this study, a total of 230 CHD patients and 263 control subjects with no reported cardiac phenotypes were recruited from Linyi People's Hospital and the Second Affiliated Hospital of Harbin Medical University, Harbin, China (Table 1). The 263 control subjects were enrolled at the Medical Examination Center of the Second Affiliated Hospital of Harbin Medical University. All these subjects had physical and electrocardiogram examinations and ultrasonic echocardiogram examination, and none of them showed any defects in the heart or other parts of the body. A written informed consent was obtained from each participant, and this work had been approved by the Ethics Committee of Harbin Medical University, consistent with the 1975 Declaration of Helsinki. Detailed records on their medical history, physical examination and chest X-ray examination, electrocardiogram, and ultrasonic echocardiogram were obtained. We deposited our data in the NIH Short Read Archive dataset, with the accession number SRP043439.

**Table 1:** Clinical characteristics of study population.

*Parameter*	*CHD*	*Control*
***Sample (n)***	230	263
***Male/Female (n)***	142/88	171/92
**Age (years)**	16.18±10.22	8.36±9.98

Data are shown as mean±SD.

### DNA analysis

Genomic DNA was extracted from peripheral blood leukocytes using standard protocols. The human *Lefty1* and *Lefty2* genes are located on 1q42.1 and are encoded by four exons. The four exons and the splicing sites of the two genes were amplified by polymerase chain reaction (PCR) with the primers shown in Table 2. PCR products were sequenced using the BigDye Terminator Cycle Sequencing kit (Applied Biosystems, Foster City, CA, USA) and the ABI 3130XL (Applied Biosystems) sequencer for mutational analysis.

**Table 2:** PCR primers used for Lefty sequence analysis.

*Gene*	*Exon*	*Forward primer*	*Reverse primer*	*Size*	*Tm*
***LEFTY1***	1	TGCCTGAGACCCTCCTGC	CCCTCACTCAGCCTCCCA	436	59.9
	2	TTTGCCCCAGAAATAGAACAGG	GACCCAGCGCCGCTTGAG	499	62.1
	3	CAACCGCACCTCCCTCAT	CATTCATTCCCACAGCACTC	513	59.2
	4	TAAATCTCCATCCCAGACGC	ACCCTCGAACACTTCAGAAACA	499	57.9
***LEFTY2***	1	CTCCCTCTTCCCTTCACCC	ACAGCCTCCCACAGAGTCCC	511	60.5
	2	GCCTGGCTGCCAGCTCAG	GACCCAGCGCCGCTTGAG	462	62.7
	3	CAACCGCACCTCCCTCATC	GCAATCGCTGGCATCCTG	570	61.7
	4	CCTCCCAGGTGCCCACTA	GGGATGGAGTAACTTGCTAA	549	56.5

### Rs2295418, Rs360057 and g.G169A *Lefty* SNP genotyping analysis and Statistical methods

**Figure 1:**
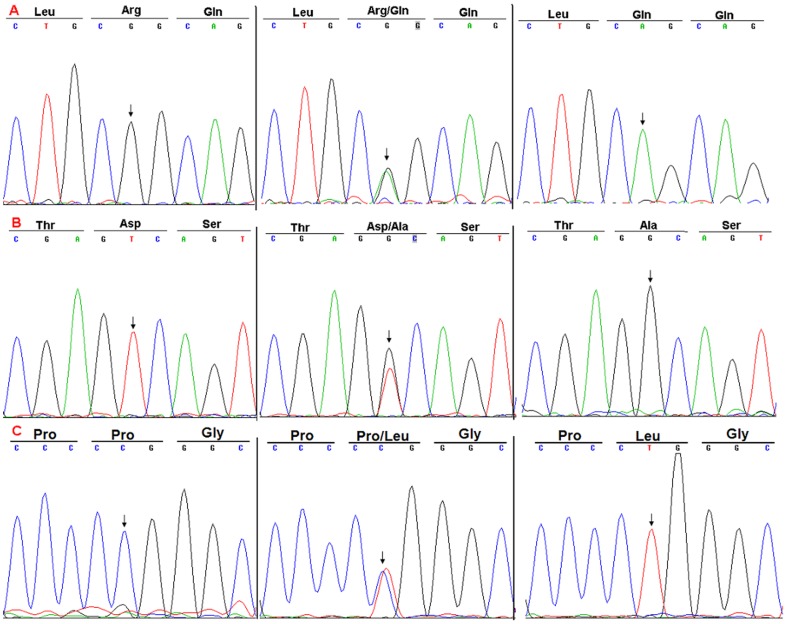
DNA sequence chromatograms of the *Lefty-1* and *Lefty -2* genes. A: g.G169A (p.Arg33Gln); B: g.A1035C-rs360057 (p.Asp322Ala); C: g.C925A-rs2295418 (p.Pro286Leu).

Genotypes of the rs2295418 and rs360057, g.G169A SNPs, within the *Lefty2* or *Lefty1* genes (Figure 1), were determined using two stage methods. We amplified rs2295418, rs360057 and g.G169A (Table 2, Lefty2exon4; Lefty1exon4 and exon1) and sequenced the PCR products to determine the genotype. The statistical analyses were conducted using Chi-Square Tests to calculate odds ratios and P value as implemented in SPSS (version 13.0). We also used online software OEGE to conduct the Hardy-Weinberg equilibrium test of the CHD and control population.

### Multiple sequence alignments

From the NCBI website (http://www.ncbi.nlm.nih.gov/), the LEFTY protein sequences of various species were obtained, and using the Vector NTI software, multiple-sequence alignments of LEFTY proteins were carried out.

## Results

### Patients

Clinical diagnosis of the recruited patients was confirmed in Linyi People's Hospital and The Second Affiliated Hospital of Harbin Medical University. There was no history of other systemic abnormalities in these CHD patients, and their mothers did not have a history of taking medicines or attracting infections during pregnancy. The 230 CHD patients contained 12 pulmonary stenosis, 14 tetralogy of Fallot, 14 patent ductus arteriosus, 22 mitral valve insufficiency, 41 atrial septal defect, 95 ventricular septal defect and 32 other complex congenital heart diseases.

### *Lefty* gene analysis

**Figure 2:**
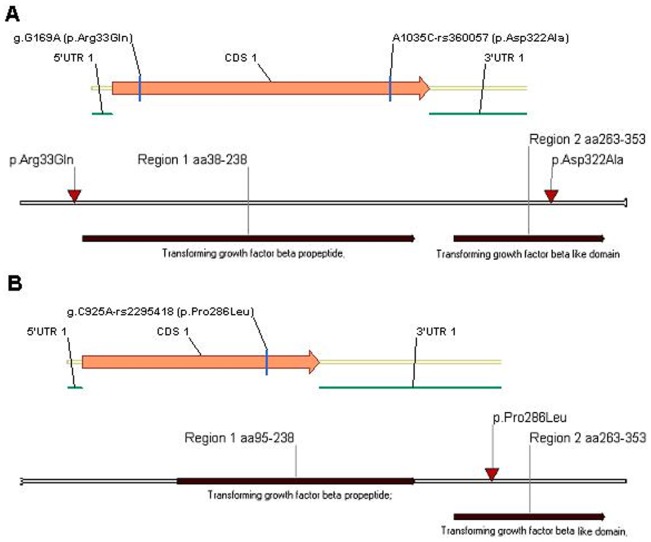
Schematic diagrams of rs2295418 and rs360057 locations within the translated region of *Lefty-2* and *Lefty -1* genes and transforming growth factor-β-like domain of the proteins. A: Lefty-1; B: Lefty-2.

We sequenced *Lefty* to test the hypothesis that germline common genetic variants in *Lefty* may confer susceptibility to CHD. We first compared the transcribed region and splicing sites of *Lefty* and found two variations in the *Lefty1* gene [g.G169A (p.Arg33Gln) and g.A1035C-rs360057 (p.Asp322Ala)] and one variation in the *Lefty2* gene [g.C925A-rs2295418 (p.Pro286Leu)] in the CHD cases (Figure 1). These variations were located within the translated region of the genes, and the g.A1035C-rs360057 and g.C925A-rs2295418 variations were located within the transforming growth factor-β-like domain of LEFTY protein (Figure 2).

### Rs2295418, Rs360057 and g.G169A Lefty SNP genotyping and Statistical analysis

To further test any possible associations between *Lefty* and CHD, we conducted SNP analyses and found that the rs2295418 (g.C925A) variant in *Lefty2* gene was obviously associated with the risk of CHD; the genotype frequency of the rs360057 (g.A1035C) variant in *Lefty1* gene was associated with the risk of CHD, but there was no statistical significance in the allele frequency. The g.G169A variant in *Lefty1* gene was not associated with the risk of CHD in the Chinese Han population (Tables 3, 4). We also conducted the Hardy-Weinberg equilibrium test for the CHD patients and controls and our results were in line with the Hardy-Weinberg equilibrium.

**Table 3:** The genotype and allele frequency of SNP rs2295418, rs360057 and g.G169A in 230 Chinese Han CHD patients and 263 non-CHD controls.

*SNP*	*Group*		*Genotype frequency (%)*			*Allele frequency (%)*	
***rs2295418***	Genotype		G/G	G/A	A/A	G	A
	CHD	230	173(75.2)	45(19.6)	12(5.2)	391(85.0)	69(15.0)
	Controls	263	223(84.8)	35(13.3)	5(1.9)	481(91.4)	45(8.6)
***rs360057***	Genotype		T/T	T/G	G/G	T	G
	CHD	230	148(64.3)	62(27.0)	20(8.7)	358(77.8)	102(22.2)
	Controls	263	167(63.5)	89(33.8)	7(2.7)	423(80.4)	103(19.6)
***g.G169A***	Genotype		G/G	G/A	A/A	G	A
	CHD	230	179(77.8)	47(20.4)	4(1.7)	405(88.0)	55(12.0)
	Controls	263	203(77.2)	56(21.3)	4(1.5)	462(87.8)	64(12.2)

**Table 4:** SNP rs2295418, rs360057 within Lefty-2 and Lefty-1 associated with the risk of congenital heart diseases in Chinese populations.

*Genotyped SNP*	*Associated gene*		*Pearson Chi-square*				*Pearson's R*			
			Value	Min count^a^	df	Asymp. Sig. (2-sided)	Value	Asymp. Std. error^b^	Approx. T^c^	Approx. Sig
***rs2295418***	LEFTY2	Genotype	8.274	7.93	2	0.016	−0.129	0.044	−2.893	0.004^d^
		Allele	9.968	53.18	1	0.002	−0.101	0.032	−3.170	0.002^d^
***rs360057***	LEFTY1	Genotype	10.069	12.60	2	0.007	−0.044	0.045	−0.966	0.334^d^
		Allele	1.001	95.64	1	0.317	−0.032	0.032	−1.000	0.318^d^
***g.G169A***	LEFTY1	Genotype	0.086	3.73	2	0.958	0.005	0.045	0.100	0.920^d^
		Allele	0.010	55.52	1	0.919	0.003	0.032	0.101	0.919^d^

a: The minimum expected count;

b: Not assuming the null hypothesis;

c: Using the asymptotic standard error assuming the null hypothesis;

d: Based on normal approximation.

### Conservation of the protein in evolution

**Figure 3:**
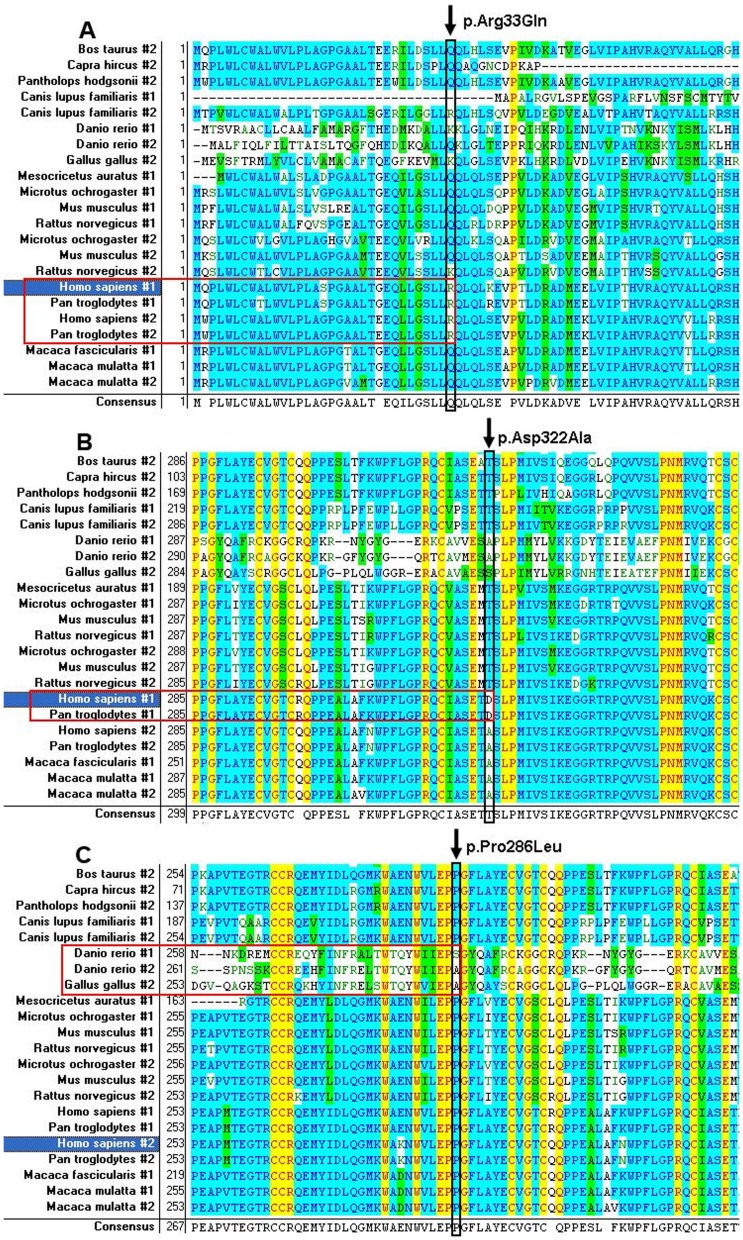
Multiple-sequence alignment of Lefty-1(#1) and -2(#2) from birds, fishes and mammals (including *Homo sapiens*, *Pan troglodytes*, *Macaca mulatta* etc.). A: p.Arg33Gln; B: p.Asp322Ala; C: p.Pro286Leu.

Comparison of the LEFTY1 and LEFTY2 protein sequences from species including birds, fishes and mammals by multiple-sequence alignment analysis showed that the 286Pro residue in LEFTY2 was highly conserved among the mammals but the 33Arg and 322Asp residues in LEFTY1 were just conserved in Chimpanzee and Humans (Figure 3).

## Discussion

In this study, we analyzed the transcribed regions and splicing sites of the *Lefty* genes in large cohorts of CHD patients and controls and found that two variants, rs2295418 (g.C925A) and rs360057 (g.A1035C), were associated with the risk of CHD in the Chinese Han population, demonstrating the involvement of the *Lefty* genes in the CHD etiology.

The formation of the human heart starts on day 18 or 19 in the mesoderm after fertilization and involves strict temporal, spatial, and sequential gene expressions. Nodal/TGF-Lefty signaling pathway acts upon gastrulation, which develops to progenitor cells of the mesoderm and endoderm [22]. In mice, the formation of mesendoderm was affected by the expression level of Nodal/TGF-Lefty signaling pathway [23], and mutations in the Nodal gene can affect the formation of primitive streak, which is formed by mesendoderm progenitor cells. The vascular systems of the mouse arise from extraembryonic mesoderm that migrate through the primitive streak to the presumptive yolk sac [24]. At later stages of embryonic development, Nodal expression initiates a series of signal transduction and induces its own and *Lefty* gene expression, and the LEFTY negatively regulates the Nodal/TGF-Lefty signaling pathway [16], [22].

We analyzed genes of the Nodal/TGF-Lefty signaling pathway, which has been demonstrated to play vital roles in mouse mesoderm differentiation and heart formation; such genes are also temporally expressed in the differentiation of the HES cells [22]. We here demonstrated that the rs2295418 (g.C925A) and the rs360057 (g.A1035C) variants in *Lefty2* and *Lefty1* genes were associated with the risk of CHD in the Chinese Han population. These nucleotides were conserved only between Chimpanzee and man among the species compared. SNP-rs1904589 within the Nodal gene, which we also analyzed in the study, was not found to be significantly associated with the risk of CHD in the population (data not show).

Of great interest, although the translated regions of the two genes are 97.18% similar in nucleotide sequence, *Lefty2* plays a more central role in the mesoderm differentiation [25], which may at least partly explain why the rs2295418 variants in *Lefty2* gene were so closely associated with the risk of CHD. In contrast to *Lefty2*, *Lefty1* seems to be less involved: although the genotype frequency of the rs360057 variant in *Lefty1* gene was apparently associated with the risk of CHD, its allele frequency was not. Further work will be needed on the Nodal/TGF-Lefty signaling pathway for their involvement in the pathogenesis of CHD at the molecular level.
